# Indoleamine 2,3-Dioxygenase (IDO) Expression Is an Independent Prognostic Marker in Esophageal Adenocarcinoma

**DOI:** 10.1155/2020/2862647

**Published:** 2020-09-22

**Authors:** Heike Loeser, Max Kraemer, Florian Gebauer, Christiane Bruns, Wolfgang Schröder, Thomas Zander, Hakan Alakus, Arnulf Hoelscher, Reinhard Buettner, Philipp Lohneis, Alexander Quaas

**Affiliations:** ^1^Institute of Pathology, University Hospital Cologne, Germany; ^2^Gastrointestinal Cancer Group Cologne, Department I for Internal Medicine, Center for Integrated Oncology, University Hospital of Cologne, Cologne, Germany; ^3^Department of General, Visceral and Cancer Surgery, University Hospital Cologne, Germany; ^4^Department I of Internal Medicine, Center for Integrated Oncology (CIO), University Hospital Cologne, Germany; ^5^Center for Esophageal and Gastric Surgery, AGAPLESION Markus Krankenhaus, Frankfurt, Germany

## Abstract

**Background:**

Indoleamine 2,3-dioxygenase (IDO) is an interferon-inducible immune checkpoint expressed on tumor-infiltrating lymphocytes (TILs). IDO is known as a poor prognostic marker in esophageal squamous cell cancer, while a positive effect was shown for breast cancer. A comprehensive analysis of IDO expression in a well-defined cohort of esophageal adenocarcinoma (EAC) is missing.

**Methods:**

We analyzed 551 patients with EAC using single-protein and multiplex immunohistochemistry as well as mRNA in situ technology for the expression and distribution of IDO on subtypes of TILs (INF-*γ* mRNA and CD4- and CD8-positive T lymphocytes).

**Results:**

IDO expression on TILs was seen in up to 59.6% of tumors, and expression on tumor cells was seen in 9.2%. We found a strong positive correlation of IDO-positive TILs, CD3-positive T lymphocytes, and INF-*γ* mRNA-producing TILs in the tumor microenvironment of EACs showing significantly better overall survival (47.7 vs. 22.7 months, *p* < 0.001) with emphasis on early tumor stages (pT1/2: 142.1 vs. 37.1 months, *p* < 0.001). In multivariate analysis, IDO is identified as an independent prognostic marker.

**Conclusions:**

Our study emphasizes the importance of immunomodulation in EAC marking IDO as a potential biomarker. Beyond this, IDO might indicate a subgroup of EAC with an explicit survival benefit.

## 1. Background

Esophageal cancer is associated with the sixth highest cancer-related mortality rate and a median survival time of 29 months [1, 2]. Although esophageal adenocarcinoma (EAC) is the fastest growing cancer in the western world, most of the therapy concepts for EAC remain largely ineffective [3]. Multimodal therapy consists of esophageal en bloc resection and perioperative radiochemotherapy; nevertheless, new therapeutic options are urgently needed to improve therapeutic concepts and prognosis in EAC.

The interaction of the tumor and its associated immune compartment is supposed to play an important role in cancer progression [4]. Mechanisms of immunosuppression in tumor microenvironment are not completely understood, although antigen loss and negative regulation by immune checkpoints are presumed to lead to dysfunction of T cells [5, 6]. Thus, identification of further immune-modulating targets is an important part of the current cancer research.

Indoleamine 2,3-dioxygenase (IDO) is an intracellular enzyme affecting T cell activity and immune tolerance. IDO expression has been detected in immune cells, stromal cells, and cancer cells and revealed relevance in cancer development and progression [7]. The enzyme catalyzes the rate-limiting step in the catabolism of local tryptophan which finally leads to anergy of effector T cells and promotion of regulatory T cells (Tregs) [8]. In several malignancies [9–11], IDO expression in either tumor cells or tumor-associated cells has been linked to adverse outcome, as demonstrated by Jia et al. for esophageal squamous cell carcinoma (ESCC) [12]. This is contrasting other studies with evidence for an improved survival in renal cell carcinoma and breast cancer [13].

The mechanism of IDO induction is not completely clear. However, it is widely accepted that interferons, particularly INF-*γ*, stimulate IDO expression in various cell types [14]. INF-*γ* is a pleiotropic cytokine supposed to play a central role in antitumor immunity with cytostatic, proapoptotic, and immune-provoking effects [15]. Besides the antitumoral effect, there is still evidence for an alternative, protumorigenic impact of *γ*-INF [16]. Interestingly, IDO induction via INF-*γ* is one mechanism presumed to contribute to an immunosuppressive tumor microenvironment in malignant melanoma and colorectal cancer [16, 17]. Recently, Rosenberg et al. analyzed the mRNA data of IDO and other checkpoint markers in The Cancer Genome Atlas (TCGA) cohort of esophageal cancer [18]. They found IDO to be related to worse prognosis in both squamous cell cancer and adenocarcinoma of the esophagus. However, both tumor entities included a relatively small number of patients (ESCC 87 and EAC 97), and according to EAC data, only gene expression analysis was performed. So, nothing is known about the protein expression of IDO in a clinical setting of neoadjuvant-treated or primary resected esophageal adenocarcinoma patients.

In the present retrospective study, we tested the hypothesis that the protein expression of the immune checkpoint IDO on immune cells is prognostic in a large cohort of EAC. Expression levels and spatial distribution of IDO, INF-*γ* and CD3 in the tumor microenvironment were therefore analyzed using immunohistochemistry and RNA BaseScope technology on tissue microarrays (TMAs) in two cohorts. Protein distribution on the different immune cells and heterogeneity were considered, and results were correlated with clinical and molecular data.

## 2. Material and Methods

### 2.1. Patients and Tumor Samples

We analyzed formalin-fixed and paraffin-embedded (FFPE) material of 551 patients with esophageal adenocarcinomas that underwent primary surgical resection or resection after neoadjuvant therapy between 1999 and 2015 at the Department of General, Visceral and Cancer Surgery, University of Cologne, Germany. The standard surgical procedure consisted of a transthoracic en bloc esophagectomy with two-field lymphadenectomy (abdominal and mediastinal lymph nodes); reconstruction was done by formation of a gastric tube with intrathoracic esophagogastrostomy (Ivor-Lewis esophagectomy) [19]. The abdominal phase was predominantly performed as a laparoscopic procedure (hybrid Ivor-Lewis esophagectomy). Technical details of this operation are described elsewhere [20–22]. Patients with advanced esophageal cancer (cT3) or presence of lymph node metastasis in clinical staging received preoperative chemoradiation (5-Fluouracil, cisplatin, 40 Gy) or chemotherapy alone. Follow-up data were available for all patients. Patient characteristics are given in Tables 1 and 2. Depending on the effect of neoadjuvant chemo- or radiochemotherapy, there is a preponderance of minor responders, defined as histopathological residual tumor of ≥10% [23]. This retrospective study was performed in accordance with the ethical standards of the ethics committee of the University of Cologne and with the 1964 Helsinki Declaration and its later amendments or comparable ethical standards. The recently published criteria for reporting recommendations for tumor marker prognostic studies (REMARK criteria) were followed in this study [24, 25].

### 2.2. Tissue Microarray (TMA)

Immunohistochemistry and RNA BaseScope analyses were performed on tissue microarrays. Construction of the TMA was described previously [26, 27]. In brief, tissue cylinders with a diameter of 1.2 mm each were punched out from selected tumor tissue blocks using a self-constructed semiautomated precision instrument and embedded in empty recipient paraffin blocks.

In the first step, we analyzed a test cohort of 165 EAC. Therefore, we built a tissue microarray (TMA) with multiple tumor spots according to the suggestions of the international immunooncology working group for assessing tumor-infiltrating lymphocytes (TILs) on solid tumors [26–29]. Up to 8 tumor spots were punched out of the tumor considering the surface and the invasion front. In the second step, we analyzed 386 additional patients to confirm our results using a single-spot TMA.

The immunohistochemical and RNA BaseScope data were statistically correlated with survival and molecular data like *TP53* mutational status and HER2/neu status.

### 2.3. Immunohistochemistry

Immunohistochemistry (IHC) was performed on TMA slides. For IDO the rabbit IgG monoclonal antibody (D5J4E; dilution 1 : 400; Cell Signaling Technology, USA) and for CD3 the rabbit monoclonal antibody (SP7; dilution 1 : 50; Thermo Fisher Scientific, USA) were used. All immunohistochemical stainings were performed using the Leica BOND-MAX stainer (Leica Biosystems, Germany) according to the protocol of the manufacturers. The evaluation of immunohistochemical expression was assessed manually by two pathologists independently (PL and HL). Discrepant results were resolved by consensus review.

### 2.4. Strategy of Evaluation

For IDO, the expression in <1% lymphocytes was defined as negative (score = 0), 1-4% of positive lymphocytes was assessed as “low positivity” (score = 1), and >4% of lymphocytes was counted as “highly positive” (score = 2). For statistical analysis, IDO-negative tumors were tested against IDO (low and high)-positive tumors.

For CD3, CD3 expression in <3 lymphocytes/mm^2^ was evaluated as negative, >3–50 lymphocytes/mm^2^ were assessed as low positive, and >50 lymphocytes/mm^2^ were defined as highly positive considering peritumoral and intratumoral distribution.

Concerning the multispot TMA, four spots of tumor surface and invasive margin each were examined. We built the average of the scores and matched the four samples to one category based on limit values: 0-0.49 = negative and 0.5-2 = positive.

### 2.5. Multiplex Immunohistochemistry

Multiplex immunohistochemistry staining was performed on a Ventana Discovery Ultra automatic staining system using TMA slides. The following primary monoclonal antibodies were used: IDO, Cell Signaling; mouse CD8 clone C8/144B, mouse CD68 clone PG-M1 (both Dako/Agilent, USA), and rabbit CD4 clone 4B12 (Roche, Switzerland, ready to use). After conjugation with an antibody-bound enzyme (horseradish peroxidase or alcalic phosphatase), detection was carried out using DISCOVERY Silver kit (IDO), DISCOVERY Yellow kit (CD68), DISCOVERY Teal kit (CD4), and DISCOVERY Purple Kit (CD8; all Ventana/Roche, Switzerland). Counterstaining was done with hematoxylin and bluing reagent.

### 2.6. RNA In Situ (RNA BaseScope)

The RNA BaseScope assay was performed according to the manufacturer's instruction. In brief, paraffin-embedded TMA blocks were cut into 5 *μ*m sections, pretreated according to extended protocol (30 minutes for pretreatments 2 and 3), digested, and hybridized at 40°C in the HybEZ oven with human INF-*γ* mRNA probe provided by Advanced Cell Diagnostics (ACD, United Kingdom, Europe). Incubation time with hematoxylin was 10 seconds. Target expression was compared to both negative (dapB) and positive (PPIB) controls. Scoring of signals was done as recommend by the manufacturer with no staining or less than one molecule per 10 cells (TILs) = score 0, 1-3 dots/cell = score 1, 4-9 dots/cell = score 2, 10-15 dots/cell = score 3, and >15 dots/cell = score 4. dapB score was 0 and PPIB score was 2. Positivity was defined as a score > 0.

### 2.7. Statistical Analysis

Clinical data were collected prospectively according to a standardized protocol. SPSS Statistics for Mac (Version 21, SPSS) was used for statistical analysis. Interdependence between stainings and clinical data were calculated using the chi-squared and Fisher's exact tests and displayed by cross-tables. Survival curves were plotted using the Kaplan-Meier method and analyzed using the log-rank test. Univariate and multivariate analyses were performed for prognostic factors of overall survival using the Cox regression model. All tests were two sided. *p* values < 0.05 were considered statistically significant.

## 3. Results

### 3.1. Clinicopathological Characteristics

**Figure 1:**
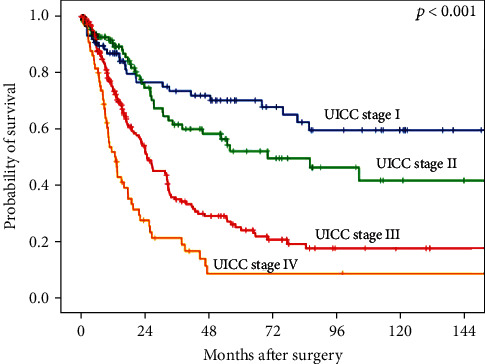
UICC stage adjusted survival for the entire patient cohort (*n* = 551).

The test cohort comprised 165 patients with EAC that underwent surgical resection. There was a male preponderance with 149 male (90.4%) and 16 female (9.6%) patients with a median age of 65.1 years (range 33-85 years) at the time of operation. To confirm our results, a single-spot TMA with additional 386 patients was analyzed, resulting in 551 patients in total. The median follow-up for the entire cohort was 57.7 months with a calculated 5-year survival rate of 26.6%. 159 (96.3%) of the samples were evaluable (Table 1) in the test cohort and 496 samples (90.0%) in the complete cohort (Table 2). The reason for noninformative cases was the absence of unequivocal cancer tissue on the TMA spot.  Figure 1 shows the survival data correlated with UICC stage in EAC.

### 3.2. Immunohistochemical Analysis of IDO

**Figure 2:**
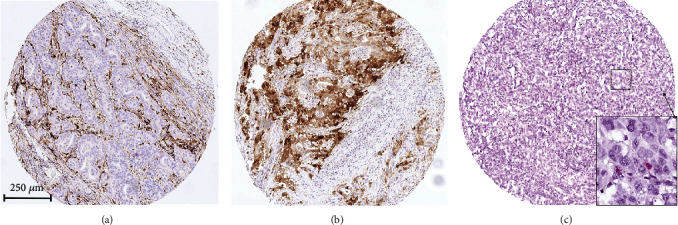
Immunohistochemistry of IDO and INF-*γ* mRNA analysis: (a) high IDO expression on tumor-infiltrating lymphocytes; (b) IDO expression of tumor cells; (c) mRNA of INF-*γ* (red signals) on tumor cells.

IDO immunostaining was localized in the cytoplasm/membrane of tumor-infiltrating lymphocytes and cancer cells (Figures 2(a) and 2(b)). In the test cohort, IDO expression on TILs (low and high positivity) was seen in 57.9% (*n* = 92) on the surface margin and 59.6% (*n* = 93) on the infiltration margin with a high correlation between the two localizations (surface and infiltration; *p* < 0.001). Cross-table analysis revealed a correlation between IDO expression and nodal-negative patients (*p* = 0.022) and low UICC stages (UICC I/II, *p* = 0.004).

On the single-spot TMA, we found IDO expression on TILs in 261 patients (52.6%). Again, a strong correlation between IDO-positive samples and early tumor stages (pT1/2) (*p* = 0.022) as well as nodal-negative patients (*p* = 0.012) was seen. IDO expression was detectable on cancer cells in 63 patients (9.2%) without any correlation with clinicopathological data.

### 3.3. Multiplex Immunohistochemistry for Subtyping of T Cells

**Figure 3:**
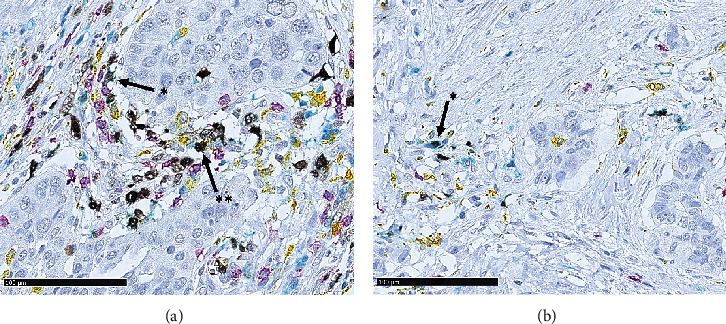
Multicolor immunohistochemistry for IDO (black signals), CD4 (teal/blue signals), CD8 (purple signals), and CD68 (yellow signals): (a) coexpression of IDO and CD4 (∗) and CD68 (∗∗); (b) coexpression with CD4 (∗).

To evaluate which subtypes of T cells expressed IDO, we performed multiplex immunohistochemistry staining on two exemplary TMAs. We correlated IDO-positive cases with the expression of CD4, CD8, and CD68 semiquantitatively (Figure 3). For IDO, a predominant coexpression with CD4 was seen, and a minor fraction demonstrated positivity for CD68. A coexpression with CD8 was not reliably detectable.

### 3.4. Immunohistochemical Analysis of CD3

The expression of CD3 was evaluated for multispot (test cohort) and single-spot TMA. CD3 distribution was predominantly seen peritumorally (*n* = 130; 78.8%). In the test cohort, roughly half of the tumors presented with high levels of CD3 (tumor surface 49.1%, infiltration margin 51.5%), which correlated well in cross-table analysis (*p* < 0.001). There was no difference between surface and infiltration margins with respect to the amounts of CD3-positive TILs.

IDO expression on TILs positively correlated with the amount CD3-positive T cells within the tumor (*p* < 0.001).

**Figure 4:**
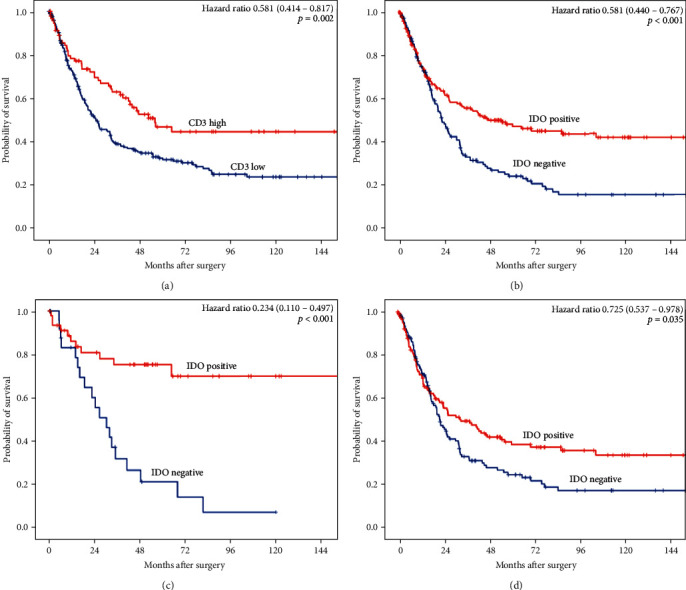
(a) High amounts of CD3-positive T cells are associated with an improved OS in esophageal adenocarcinoma. (b) Tumors with IDO-positive TILs show better median overall survival of 47.7 months in IDO-positive tumors compared to a median OS of 22.7 months for IDO-negative tumors. (c) In the pT1/2 group, patients with IDO-positive expression have a calculated average OS of 142.1 months (median not reached) compared to an average OS of 37.1 months (median OS 30.5 months), *p* < 0.001. (d) The survival difference remains significant also in the subgroup of pT3/4 tumor stages with median OS of 33.3 months for IDO-positive tumors and 22.1 months for IDO-negative tumors (*p* = 0.035).

High levels of CD3-positive TILs are associated with an improved overall survival (OS) compared to CD3-poor tumors considering the single-spot TMA of 551 patients (496 patients analyzable; *p* = 0.002; Figure 4(a)).

### 3.5. RNA BaseScope Analysis of INF-*γ*

Within the test cohort, correlation between INF-*γ* and IDO-positive TILs revealed a strong correlation in both compartments, surface and infiltration zones, respectively (*p* < 0.0001) (Figure 2(b)).

### 3.6. Correlation with Molecular Markers

*TP53* mutation and HER2 amplification/expression status was available for 356 patients. There was no correlation between the remaining histopathological parameter and *TP53* mutational or HER2/neu status. Within the IDO-positive group, 117 patients showed a *TP53* mutation (60.6%) and 76 patients (39.6%) were *TP53* wild-type tumors (*p* = 0.210). Similar results were found for HER2/neu amplification. In tumors with high IDO expression, 26 patients showed HER2/neu amplification (13.8%), but a correlation via cross-table analysis did not reveal a significant association between IDO and HER2/neu amplification (*p* = 0.116).

### 3.7. Expression of IDO on TILs Is Prognostic in EAC

In the test cohort of 165 patients, no statistically significant overall survival difference was detectable for IDO-expressing tumors, although a correlation with nodal-negative tumors and low UICC stages in IDO-positive samples was seen. However, there was a trend towards improved overall survival (OS) in patients with IDO expression.

On the single-spot TMA, tumors with IDO-expressing TILs showed an improved median OS (47.7 months (95% confidence interval (CI) 20.9-73.8 months)) compared to IDO-negative patients (median OS 22.7 months (95% CI 18.8-26.6 months), *p* ≤ 0.001) (Figure 4(b)). The hazard ratio was 0.581 (95% CI 0.440-0.767, *p* < 0.001) for patients with IDO expression.

Subgroup analyses revealed a particularly pronounced difference in OS in the group of pT1/2 stage patients. Within this group of lower tumor stages, IDO-positive patients reached a calculated average OS of 142.1 months (median not reached, average 95% CI 115.2-168.9 months) compared to an average OS of 37.1 months (95% CI 23.6-50.7 months, median OS 30.5 months (95% CI 19.9-41.1 months, *p* < 0.001)) (Figure 4(c)). However, the survival difference remains significant also in the subgroup of higher tumor stages (pT3/4) with comparable median OS values to the entire patient cohort. In the pT3/4 group, the median OS for IDO-positive patients was 33.3 months (95% CI 17.9-48.7 months) and 22.1 months (95% CI 17.9-26.3 months, *p* = 0.035) for IDO-negative patients (Figure 4(d)).

In a multivariate Cox regression analysis, IDO expression on TILs and the histopathological parameters pT and pN stages were independent prognostic markers (Table 3).

### 3.8. Impact of Neoadjuvant Therapy

216 (43.5%) patients received neoadjuvant therapy, whereas 280 (56.4%) patients primarily underwent surgical resection. The prognostic impact of IDO on OS was independent on whether neoadjuvant treatment was administered or not. IDO expression remains a positive prognostic marker in both patient groups with primary surgery and surgery with neoadjuvant treatment. Patients who underwent primary surgery without any kind of neoadjuvant treatment with the presence of IDO expression showed a median OS of 104.6 months (95% CI 50.1–159.2 months) while IDO-negative patients showed an OS of 25.4 months (95% CI 13.5–37.3 months, *p* = 0.005). Similar results were found for the group of patients after neoadjuvant treatment; again, IDO serves as a significant prognostic marker (*p* = 0.041) with a median OS of 30.8 months (95% CI 12.0–49.6 months) in IDO-positive patients compared to a median OS of 22.4 months (95% CI 18.5–26.3 months) in IDO-negative patients.

## 4. Discussion

Here, we report the expression of the immune checkpoint protein IDO on tumor-associated inflammatory cells in a large and well-characterized cohort of 551 patients with EAC. We evaluated the level of heterogeneity and distribution of IDO-positive TILs within the tumor. IDO expression on TILs was a strong and statistically independent prognostic biomarker for an improved overall survival in EAC. IDO expression correlated significantly with low UICC stages (I/II) and nodal-negative status (pN-). Furthermore, we found a strong correlation with INF-*γ* expression and the number of CD3-positive T cells within the tumors. In multicolor immunohistochemistry, we demonstrated a predominant coexpression of IDO with CD4-positive T cells. No correlation of IDO expression on TILs with important molecular alteration markers like *TP53* mutational status and HER2/neu amplification was seen.

For the test cohort of 165 patients, we built a multispot TMA considering two different tumor localizations (surface and infiltration margins) proving low heterogeneity within the 4 spots of one tumor localization and a consistent expression pattern between surface and infiltration margins, respectively. Thus, the expression of IDO in randomly taken EAC samples by endoscopic tumor biopsy is most likely representing overall tumor IDO expression. Furthermore, the absence of significant heterogeneity was one reason to evaluate IDO expression on a single-spot TMA with 386 additional patients (551 in total) to confirm our results.

To the best of our knowledge, we are the first to evaluate IDO protein expression in EAC. Rosenberg et al. analyzed IDO mRNA expression using TCGA data of squamous cell cancer and adenocarcinomas of the esophagus correlating other checkpoint markers like PD-L1 and CTLA4 [18]. Overall, they found high IDO mRNA levels being associated with worse patient survival. However, the additionally performed IDO protein expression (IHC) in 93 patients with ESCC did not correlate with survival. For EAC, their results are solely based on mRNA data.

Within our cohort, IDO protein expression on TILs was an independent prognostic biomarker within all tumor stages indicating a tremendous survival benefit especially in pT1/2 tumor stages. Since we did not detect heterogeneity in the expression pattern, high levels of IDO expression on TILs in endoscopic biopsies could probably define a subgroup of patients with a favorable prognosis, possibly with no further benefit of radiochemotherapy in this group. This could be particularly interesting for patients in advanced tumor stages with an extremely reduced expectancy of life avoiding an aggressive therapy concept with reduced benefit. Neoadjuvant treatment did not influence the prognostic effect of IDO on overall survival in this cohort of EAC. In a mouse model, IDO was shown to increase after radiotherapy [30]. In non-small-cell lung cancer (NSCLC), there is evidence that radiotherapy indeed reduces IDO activity during therapy but increases posttherapeutically with worse prognosis in NSCLC [31]. However, in that study, IDO was not analyzed directly in the tumor tissue, but kynurenine serum levels were measured as an indicator of IDO activity.

Physiologically, IDO protein catalyzes the elimination of the essential amino acid tryptophan [32]. The resulting metabolites (l-kynurenine, l-hydroxykynurenine, 3-hydroxyanthranilic acid, quinolinic acid, and picolinic acid) were initially considered to protect the host from infections but have recently been recognized to provide regulatory effects on the inflammatory microenvironment [33]. Accumulating metabolites are supposed to cause immunosuppression by the activation of regulatory T cells, apoptosis of T effector cells, and inhibition of T cell proliferation [8, 33]. Immunosuppressive characteristics of IDO have therefore accounted for the establishment of IDO inhibition in clinical trials to encourage immune response against the tumor [34]. Even Opitz et al. describe the importance of tryptophan metabolism in targeted therapy concepts in a recently published study, although they conclude that IDO inhibition has failed as a sufficient therapy concept until today [35]. Nevertheless, several studies describing an adverse influence of IDO on patients' clinical outcome refer comprehensibly to these effects of an elevated IDO expression [10, 11, 36].

However, and in opposite to these findings, we clearly demonstrate not only a favorable impact of IDO expression on TILs on overall survival in a large cohort of 551 patients with EAC but also IDO to be an independent marker for prognosis. This is absolutely in common with former research, e.g., a recent study by Patil et al. [37] examined IDO expression in gastric adenocarcinoma and found comparable results concerning the amount of IDO-positive samples (58%; our test cohort: 57.9% and 59.6%, respectively) and a favorable prognostic impact of stromal IDO expression. An elevated IDO expression is further linked to an improved overall survival in breast cancer, renal cell carcinoma, and cervical cancer [38–40]. Therefore, alternative effects of IDO expression on cancer progression have been discussed. For instance, Soliman et al. assumed that a local decrease of tryptophan might lead to metabolic growth disadvantage for tumor cells [41]. Moreover, Riesenberg et al. [40] considered toxic metabolites of tryptophan elimination to damage tumor cells and found a significantly decreased proliferation in tumor cells exposed to IDO-positive microvessels. We further hypothesized that overexpression of regulatory proteins in the immune compartment of a tumor could be part of a generally elevated immune response of particularly immunogenic tumor biology. IDO is activated by INF-*γ*, and as we could point out by using RNA BaseScope technology, IDO-positive tumors were enriched with INF-*γ*-positive inflammatory cells in the tumor microenvironment. It is therefore thinkable that the favorable outcome of IDO-positive tumors is driven by the inflammatory microenvironment but not the immune checkpoint itself, which is rather representing a subsequent regulatory counterpart in that reaction. Concordant to this, previous research on colorectal cancer found immune checkpoint expression of PD-1, PD-L1, CTLA-4, LAG-3, and IDO as a counterbalancing part of highly inflamed tumors (MSI unstable) [42].

Apart from IDO expression on inflammatory cells, we additionally found expression on tumor cells in 9.2%. IDO expression has been previously described in various cell types, including endothelial cells, mesenchymal stromal cells, fibroblasts, and various myeloid-derived antigen-presenting cells such as DCs and macrophages, as well as tumor cells [8]. Different results concerning the influence of IDO on tumor progression could therefore be dependent on the expressing cell type. In cervical cancer, Heeren et al. found differing serum levels of the immunosuppressive tryptophan metabolite l-kynurenine dependent on whether IDO is expressed on tumor cells (high levels of l-kynurenine) or immune cells (low levels of l-kynurenine) [39]. Riesenberg et al. further concluded that a selective enhancement of IDO expression in endothelial cells, but not in tumor cells, reduces tumor progression in renal cell carcinoma [40]. Therefore, our findings of a beneficial effect of IDO displayed exclusively on inflammatory cells underline the importance of an individual evaluation of IDO expression patterns for tumor cells versus stromal/inflammatory cells.

Still, open questions concerning the interaction of IDO and cancer growth need to be answered. For example, the significant decrease of IDO expression in advanced tumor stages remains cryptic. We assume that there is an interaction between immune checkpoint expression of the tumor microenvironment and invasive tumor growth. However, it is not clear whether overexpression of immune regulatory proteins negatively influences local tumor growth, or changes in tumor biology in advanced tumor stages might be responsible for a decreased level of checkpoints. Furthermore, the downstream metabolites of the tryptophan catabolism are known to influence IDO expression and activity and need to be considered in targeted therapy concepts [35].

Our study has strengths and limitations. We analyzed two independent and well-characterized cohorts of EAC. Furthermore, we identified an expression of IDO on inflammatory and tumor cells and discussed potential pro- and antitumoral effects. However, the study is retrospective and a selection bias cannot be excluded. We were not able to include patients who received neoadjuvant treatment and showed a complete tumor response or those with advanced tumors that were not eligible for surgical therapy. Beyond that, functional conclusions concerning biological mechanisms of IDO are not feasible on formalin-fixed material; therefore, future studies have to clarify the potential of IDO as a predictive biomarker in EAC.

## 5. Conclusions

In summary, our study describes the rate of IDO expression on TILs in EAC and demonstrates a strong and statistically independent positive prognostic effect in a very large group of EACs for the first time. IDO expression correlates significantly with low UICC stages (I/II) and negative lymph node status. However, prospective studies need to confirm our results. Since we find a favorable effect of IDO expression on overall survival in EAC, we assume that IDO interaction with tumor cells might be more complex than anticipated. Particularly, the IDO-expressing cell type as well as the metabolites of tryptophan catabolism might influence the effectiveness of future clinical trials investigating antibody-based IDO blockade in EAC.

## Figures and Tables

**Table 1:** Patient characteristics and IDO expression results on the test cohort (*n* = 165; 159 analyzable).

			IDO expression surface margin					IDO expression infiltration margin				
			Negative		Positive		*p* value	Negative			Positive	*p* value
	No.	%	No.	%	No.	%		No.	%	No.	%	
Sex												
Female	16	10.1%	5	31.3%	11	68.7%	0.430	5	33.3%	10	66.7%	0.783
Male	143	89.9%	62	43.4%	81	56.6%		58	41.1%	83	58.9%	
Age group												
<65 years	70	44.3%	31	44.3%	39	55.7%	0.746	31	45.6%	37	54.4%	0.248
>65 years	88	55.7%	36	77.0%	52	23.0%		31	35.6%	56	64.4%	
Tumor stage												
pT1	46	29.1%	13	28.3%	33	71.7%	0.070	13	29.5%	31	70.5%	0.200
pT2	29	18.4%	12	41.4%	27	58.6%		11	37.9%	18	62.1%	
pT3	82	51.9%	41	50.0%	41	50.0%		37	45.7%	44	54.3%	
pT4	1	0.6%	1	100%	0	0.0%		1	100%	0	0.0%	
Lymph node metastasis												
pN0	60	38.0	17	23.3%	43	71.75	0.022	20	33.9%	39	66.1%	0.154
pN1	71	44.9	34	47.9%	37	52.1%		26	37.0%	43	62.3%	
pN2	12	7.6	6	50.0%	6	50.0%		7	58.3%	5	41.7%	
pN3	15	9.55	10	66.6%	5	33.3%		9	60.0%	6	40.0%	
UICC stage												
I	41	26.1%	21	51.2%	20	48.8%	<0.001	23	57.6%	17	42.5%	0.045
II	21	13.4%	15	71.4%	6	28.6%		14	66.7%	7	33.3%	
III	75	47.8%	69	92.0%	6	8.0%		60	78.9%	16	21.1%	
IV	20	12.7%	17	85.9%	3	15.0%		17	85.0%	3	15.0%	

**Table 2:** Patient characteristics of the entire cohort, IDO expression results (*n* = 551; 496 patients analyzable).

	IDO expression single spot				
	Negative		Positive		*p* value
	No.	%	No.	%	
Sex					
Female	31	53.4%	27	46.6%	0.331
Male	204	46.6%	234	53.4%	
Age group					
<65 years	132	51.0%	127	49.0%	0.166
>65 years	105	44.5%	132	55.5%	
pT1	13	26.8%	52	73.2%	0.004
pT2	27	49.1%	28	50.9%	
pT3	179	51.1%	171	48.9%	
pT4	9	50.0%	9	50.0%	
pN0	74	38.1%	120	61.9%	<0.001
pN1	79	44.6%	98	55.4%	
pN2	40	64.5%	22	35.5%	
pN3	40	65.6%	21	34.4%	
Neoadjuvant treatment					
Yes	90	41.7%	126	58.3%	0.029
No	145	51.8%	135	48.2%	
UICC stage					
I	34	33.7%	67	66.3%	<0.001
II	44	38.9%	69	61.1%	
III	118	57.3%	88	42.7%	
IV	37	50.0%	37	50.0%	

**Table 3:** Multivariate Cox regression model; HR = hazard ratio.

	Hazard ratio	95% confidence interval		*p* value
		Lower	Upper	
Sex (male vs. female)	1.557	0.863	2.807	0.141
Age group (<65 vs. >65 years)	1.351	1.01	1.807	0.043
Tumor stage (pT1/2 vs. pT3/4)	1.429	0.916	2.229	0.116
Lymph node metastasis (pN0 vs. pN+)	2.987	2.105	4.239	0.022
CD3 (low vs. high)	0.666	0.459	0.966	0.032
IDO on TILs (negative vs. positive)	0.729	0.537	0.991	0.044

## Data Availability

All data of the study are available whenever requested.
